# *Shigella* Manipulates Host Immune Responses by Delivering Effector Proteins with Specific Roles

**DOI:** 10.3389/fimmu.2015.00219

**Published:** 2015-05-07

**Authors:** Hiroshi Ashida, Hitomi Mimuro, Chihiro Sasakawa

**Affiliations:** ^1^Division of Bacterial Infection Biology, Institute of Medical Science, University of Tokyo, Tokyo, Japan; ^2^Division of Bacteriology, Department of Infectious Diseases Control, International Research Center for Infectious Diseases, The Institute of Medical Science, University of Tokyo, Tokyo, Japan; ^3^Nippon Institute for Biological Science, Tokyo, Japan; ^4^Medical Mycology Research Center, Chiba University, Chiba, Japan

**Keywords:** *Shigella*, effector, inflammation, innate immunity

## Abstract

The intestinal epithelium deploys multiple defense systems against microbial infection to sense bacterial components and danger alarms, as well as to induce intracellular signal transduction cascades that trigger both the innate and the adaptive immune systems, which are pivotal for bacterial elimination. However, many enteric bacterial pathogens, including *Shigella*, deliver a subset of virulence proteins (effectors) via the type III secretion system (T3SS) that enable bacterial evasion from host immune systems; consequently, these pathogens are able to efficiently colonize the intestinal epithelium. In this review, we present and select recently discovered examples of interactions between *Shigella* and host immune responses, with particular emphasis on strategies that bacteria use to manipulate inflammatory outputs of host-cell responses such as cell death, membrane trafficking, and innate and adaptive immune responses.

## Introduction

**Figure 1:**
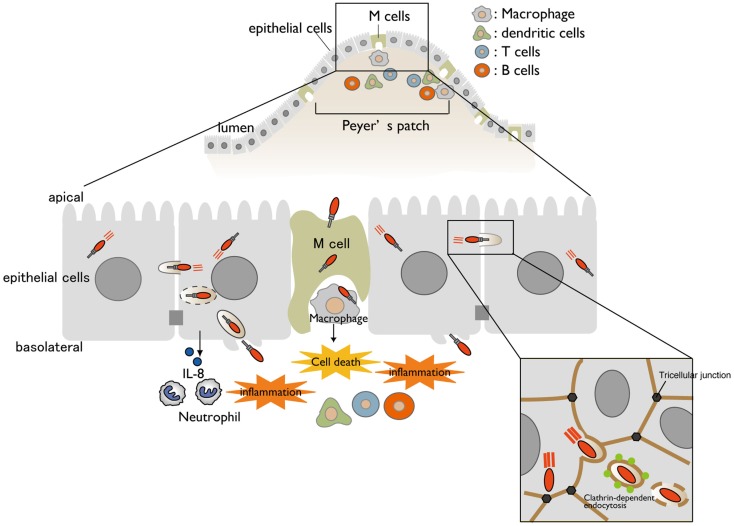
**A model for *Shigella* infection of the intestinal epithelium**. A schematic representation of *Shigella* infection. Bacterial invasion and multiplication within macrophages and epithelial cells cause subsequent massive inflammatory colitis, termed as shigellosis. Cell-to-cell movement of *Shigella* occurs at tricellular junctions via clathrin-dependent endocytosis.

*Shigella* is a causative agent of bacillary dysentery, which ultimately leads to severe bloody and mucous diarrhea (shigellosis). Most cases of shigellosis occur in developing countries and affect children under 5 years old. Although antibiotics are the standard care for shigellosis patients, antibiotic-resistant bacterium is becoming common. Therefore, it is urgently necessary to develop a safe and effective *Shigella* vaccine. *Shigella* have neither adherence factor nor flagella, but they are capable of efficiently invading the intestinal epithelium. *Shigella* injects a subset of effectors (secreted virulence proteins) via a type III secretion system (T3SS) (protein delivery system) into host cells, allowing the bacterium to invade, multiply within the intestinal epithelium, and subvert cellular and immune functions during bacterial internalization (1, 2). When *Shigella* cells are ingested via the oral route, the bacteria move down to the colon and rectum, and then preferentially enter the M cells overlying the follicle-associated epithelium of the Peyer’s patches (3, 4) (Figure 1). Once the bacteria are endocytosed by the M cells, they are transcytosed toward the M cell pocket, where resident macrophages receive the bacteria. However, *Shigella* can disrupt the vacuolar membranes, disseminate into the cytoplasm, and multiply therein (5). Bacterial multiplication within the macrophages results in massive inflammatory cell death (6) (Figure 1). Meanwhile, *Shigella* cells that are released from dying macrophages subsequently enter the surrounding epithelium via the basolateral surface. Upon epithelial cell contact, the bacteria deliver a subset of T3SS effector proteins that trigger actin rearrangement, promoting bacterial uptake (7). Next, the *Shigella* cells are surrounded by a vacuolar membrane, but the bacteria rapidly disrupt this membrane and disseminate into the cytoplasm. As *Shigella* proliferates within the cytoplasm, it moves by inducing actin polymerization at one pole of the bacterium, providing the propulsive force required for inter- or intracellular movement (8–10). Intriguingly, *Shigella* cell-to-cell movement preferentially occurs at epithelial tricellular junctions, where three cells meet (Figure 1). At these positions, bacteria-containing pseudopodia are engulfed by neighboring cells via a clathrin-dependent endocytic pathway, resulting in dissemination of *Shigella* (11). By repeating these processes, the bacteria efficiently multiply by constantly renewing their replicative compartment. Thus, multiple infectious events during *Shigella* infection, including macrophage cell death, invasion of and multiplication within epithelial cells, cell-to-cell spreading, demise of the host epithelium, and alteration of the host inflammatory response, are major pathogenic events that lead to shigellosis (2) (Figure 1).

## Cell Death

Host-cell death in response to microbial infection is an intrinsic immune defense stratagem against microbial intrusion. The sacrifice of infected cells plays a pivotal role in clearance of damaged cells, elimination of pathogens, local confinement of tissue damage and inflammation, and presentation of bacteria-derived antigens to the adaptive immune system (12). Cell death induced by bacterial infection can be classified into at least three types, depending on the type of cell and stage of infection: apoptosis, necrosis, and pyroptosis. Apoptosis is a non-inflammatory programed cell death triggered by the mitochondria-mediated pathway and receptor-mediated pathway, which eventually induce caspase activation (caspase-2, -3, -6, -7, -8, -9, and -10), chromatin condensation, cell shrinkage, plasma membrane blebbing, and cytoplasm retained in apoptotic bodies. On the other hand, necrosis is an inflammatory form of cell death characterized by cell swelling, membrane rupture, and intracellular content leakage. Pyroptosis is pro-inflammatory, lytic, and programed cell death that is accompanied by activation of caspase-1 or caspase-11 (human homologs: caspase-4/5) inflammasomes, leading to the production of IL-1β and IL-18.

When bacteria invade and multiply within host cells, they release bacterial components [e.g., lipopolysaccharide (LPS) and peptidoglycan (PGN)] and T3SS components, and further cause infection-associated cellular damage. These are recognized as pathogen-associated molecular patterns (PAMPs) and danger-associated molecular patterns (DAMPs) by recognition receptors, such as toll-like receptors (TLRs), nucleotide-binding oligomerization domain-like receptors (NLRs), AIM2-like receptors (ALRs), and RIG-like receptors, and trigger host immune responses against bacterial infection. Upon recognition of these PAMPs and DAMPs, a subset of NLRs (e.g., NLRP1, NLRP3, and NLRC4) and ALRs (e.g., AIM2) form inflammasomes, which are multi-protein signaling complexes composed of NLR/ALR, the adaptor protein ASC, and inflammatory caspase, such as caspase-1 (canonical inflammasomes) and caspase-11 (non-canonical inflammasomes) (13, 14). Inflammasome activation ultimately results in release of pro-inflammatory cytokines (IL-1β and IL-18) and induction of pro-inflammatory lytic cell death (pyroptosis).

**Figure 2:**
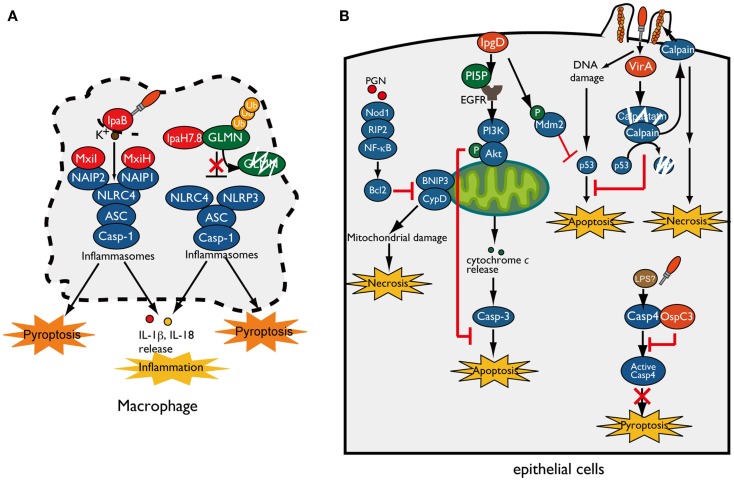
***Shigella* manipulates host-cell death**. **(A)** *Shigella* invasion and replication in macrophages trigger NLRC4/NLRP3-inflammasomes activation and pyroptotic cell death. The *Shigella* T3SS rod component MxiI and needle protein MxiH trigger NAIP2- and NAIP1-dependent NLRC4-inflammasome activation, respectively, whereas T3SS effector IpaB-mediated potassium influx triggers NLRC4-inflammasome activation and ultimately induces pyroptosis. IpaH7.8 ubiquitinates GLMN, and undergoes proteasome-dependent degradation, resulting in NLRP3/NLRC4-inflammasome activation and pyroptosis. **(B)** *Shigella* counteracts mitochondrial damage-dependent necrosis through BNIP3 and CypD by activating the PGN/Nod1/RIP2/NF-κB/Bcl2 pro-survival pathway. Furthermore, *Shigella* prevents epithelial cell death by delivering an array of T3SS effectors. PI5P, generated by IpgD, promotes EGFR activation, which contributes to sustained activation of the PI3K–Akt survival pathway. IpgD and VirA target Mdm2 and calpain, respectively, to prevent p53-dependent apoptosis. OspC3 binds to the caspase-4 subunit and inhibits its activation, thereby blocking caspase-4-dependent inflammatory cell death.

When *Shigella* invade and multiply within macrophages, they rapidly induce pyroptotic cell death accompanied by NLRP3- or NLRC4-inflammasome activation, leading to IL-1β and IL-18 secretion (15–18). T3SS needle or rod components indirectly activate NLRC4 inflammasomes through members of the NAIP subfamily of NLRs, which act as direct pathogen recognition sensors and determine the specificity of the NLRC4-inflammasome for various bacterial ligands. Recent reports have revealed that human NAIP and mouse NAIP1 bind to *Shigella* T3SS needle protein MxiH (19, 20). In addition, NAIP2 binds to the T3SS inner rod component MxiI (16, 21) (Figure 2A). Although *Shigella* lacks flagella, NAIP5 and NAIP6 specifically activate the NLRC4-inflammasome in response to other bacterial flagellins (22–25). Once these NAIP proteins bind to their ligands, they bind to NLRC4 and induce NLRC4-inflammasome activation and pyroptosis. In addition to bacterial T3SS components, cellular damage caused by *Shigella* also triggers inflammasome activation and pyroptosis. As *Shigella* cells multiply within macrophages, the T3SS effector IpaB assembles an ion channel within cell membrane to allow for potassium influx, which is recognized by the NLRC4-inflammasome and ultimately triggers pyroptosis (26) (Figure 2A).

As described above, because pyroptosis is accompanied by inflammation that would limit bacterial infection, it had not been unclear whether pyroptosis is beneficial for bacterial infection (27). However, a recent study showed that induction of inflammasome activation and pyroptosis in infected macrophages is a *Shigella* strategy that can promote bacterial survival and dissemination. Suzuki et al. showed that *Shigella* induces rapid macrophage pyroptosis via IpaH7.8, an IpaH family effector, mediated NLRP3- and NLRC4-dependent inflammasome activation (28) (Figure 2A). IpaH family effectors, which have a novel E3 ubiquitin ligase activity, are widely conserved among Gram-negative bacterial pathogens, including *Shigella*, *Salmonella*, *Yersinia*, and *Pseudomonas* spp. (29). IpaH7.8 targets GLMN (glomulin/flagellar-associated protein 68), a Cullin-RING E3 ligase inhibitor, for ubiquitination and undergoes proteasome-dependent degradation (Figure 2A) (28). Because GLMN acts as a negative regulator of NLR inflammasomes and pyroptosis, degradation of this factor induces inflammasome activation and pyroptosis. Consistent with results obtained *in vitro*, mice intranasally infected with *Shigella* WT or Δ*ipaH7.8/WT* complement strains induce more severe inflammatory responses and elevated numbers of colonized bacteria relative to Δ*ipaH7.8* or Δ*ipaH7.8/CA* E3 ligase-deficient mutant complemented strains (28). Therefore, IpaH7.8-mediated macrophage cell death is prerequisite for allowing bacteria to escape from macrophages, further enter surrounding epithelial cells, and spread to neighboring cells.

## Epithelial Cell Death

When *Shigella* invade and multiply within epithelial cells, the cells generate an early genotoxic stress, mitochondrial damage, oxidative stress, and recognize PAMPs and DAMPs, which could induce several types of cell death as part of the host defense system aimed at terminating bacterial infection. However, in contrast to macrophage infection, *Shigella* seems to prevent epithelial cell death until the bacteria have fully multiplied, because it prefers these cells as replicative niche, spread to neighboring cells, and evasion of immune cells (30) (Figure 2B). To support this notion, *Shigella* has several countermeasures that inhibit epithelial cell death: (i) prevention of mitochondrial damage, (ii) activation of cell survival signaling [e.g., phosphoinositide-3 kinase (PI3K)-Akt and transcription factor nuclear factor κB (NF-κB)], and (iii) prevention of caspase activation. For example, *Shigella* prevents necrotic cell death mediated by mitochondrial damage (through BNIP3 and CypD) by activating the Nod1–RIP2–NF-κB–Bcl-2 pro-survival pathway (31) (Figure 2B). When *Shigella* invades epithelial cells, it delivers the T3SS effector IpgD (a homolog of *Salmonella* SopB), a phosphoinositide phosphatase that converts phosphatidylinositol 4,5-bisphosphate (PIP2) into phosphatidylinositol 5-phosphate (PI5P), at the bacterial entry site (32). Because PI plays pivotal roles in actin cytoskeleton rearrangement, elevated levels of PI5P at the plasma membrane promotes bacterial invasion. Furthermore, IpgD-mediated PI5P is an important factor involved in cell survival (33). At this step, the PI5P generated by IpgD contributes to epidermal growth factor receptor (EGFR) activation, which sustains the PI3K/Akt pro-survival pathway and thereby contributes indirectly to augmentation of pro-survival signaling (33, 34) (Figure 2B). A recent report showed that the early stage of *Shigella* infection induces genotoxic stress in epithelial cells, followed by p53 pro-apoptotic signaling activation and induction of apoptosis; however, *Shigella* promotes p53 degradation and antagonizes the early stage of cell death by delivering two T3SS effectors, IpgD and VirA (Figure 2B) (35). As described above, IpgD promotes Akt activation, which in turn phosphorylates and stabilizes the downstream E3 ubiquitin ligase Mdm2. Activated Mdm2 targets p53 for ubiquitination and leads to proteasome-dependent degradation, thereby inhibiting pro-apoptosis signaling by p53. Furthermore, VirA promotes further degradation of p53 by activating calpain protease, which is also important for bacterial invasion. VirA binds to the calpain inhibitor calpastatin and promotes its degradation, resulting in degradation of p53 by activated calpain and blocking the p53 pro-apoptotic signaling pathway. Although VirA-mediated calpain activation promotes bacterial entry and prevents the early stage of apoptosis, sustained calpain activation ultimately induces necrosis and restricts bacterial proliferation (35). In addition, *Shigella* exploits calpain activation and mitochondrial function to suppress the innate immune response without inducing host-cell death (see below) (36). These results indicate that *Shigella* deploys a sophisticated strategy that controls the delicate balance of host-cell death until *Shigella* has succeeded in primary colonization and proliferation within epithelial cells.

Recent reports have shown that non-canonical caspase-4/-11 inflammasome activation is an essential host defense mechanism of epithelial cells against enteric bacterial pathogens such as *Shigella*, *Salmonella*, and enteropathogenic *Escherichia coli* (EPEC). Caspase-4/-11 directly binds to cytoplasmic LPS of Gram-negative bacterial pathogens and activates caspase-4/-11-mediated inflammasomes and pyroptosis, ultimately inducing epithelial cell shedding to eliminate infected cells (37–39). However, *Shigella* antagonizes caspase-4-dependent inflammatory cell death by delivering the T3SS effector OspC3 and promoting epithelial infection (37). OspC3 deploys a unique mechanism that specifically targets and inactivates caspase-4, but not caspase-1 or mouse caspase-11. The C-terminal ankyrin-repeat (ANK) region of OspC3 interacts with the p19 subunit of caspase-4 and prevents its activation by inhibiting p19 and p10 dimerization (Figure 2B). In human epithelial cell lines, the *Shigella* Δ*ospC3* mutant induces early caspase-4-dependent pyroptotic cell death and increased cytokine production relative to that of WT *Shigella*. Of note, the *Shigella* Δ*ospC3* mutant also exhibited severe mucosal cell death and a reduction in the number of colonizing bacteria in a guinea pig rectal infection model, indicating the importance of OspC3-mediated cell death inhibition for bacterial infection (37). Thus, *Shigella* delivers a subset of T3SS effector proteins that delay epithelial cell death until the bacterial cells have fully replicated and further disseminated into surrounding cells.

## Autophagy

Autophagy is an essential cellular catabolic process, which targets proteins, organelles, and large protein aggregates by sequestering deleterious cargos within a double-membrane compartment, the autophagosome. Autophagy also plays a pivotal role as a part of the innate immune system, by acting as a cytosolic sensor to recognize DAMPs and PAMPs, and as an “executioner” that engulfs bacteria in autophagosomes that fuse with lysosomes, ultimately destroying bacteria within lysosomal compartments.

**Figure 3:**
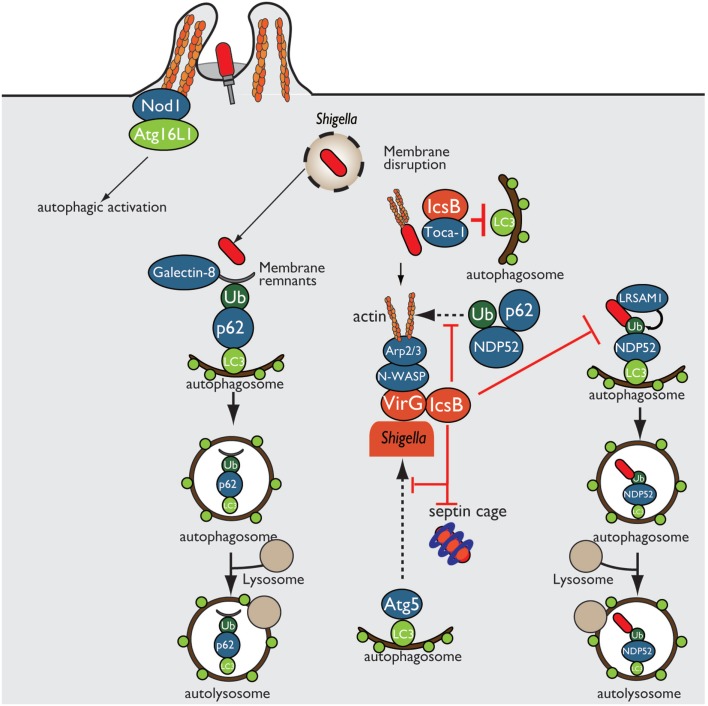
***Shigella* prevents autophagic recognition**. *Shigella* prevents autophagic clearance by evading autophagic recognition. *Shigella* delivers the T3SS effector IcsB, which binds to the bacterial outer membrane protein VirG and prevents Atg5–VirG interaction and ubiquitin recruitment, thereby evading autophagic recognition. IcsB also recruits Toca-1, and prevents the recruitment of LC3 to around intracellular bacteria.

*Shigella* enter epithelial cells, disseminate into the cytosol by disrupting the surrounding phagosomal membrane, and move into adjacent cells by inducing actin polymerization at one bacterial pole. In *Shigella* invasion of epithelial cells, Nod1 and Atg16L1 are recruited to the plasma membrane beneath the *Shigella* entry site and subsequently trigger autophagy (40). Furthermore, host-cell vacuolar membrane remnants generated by *Shigella* are recognized as DAMPs by galectin-8, and these remnants are also polyubiquitinated, followed by the recruitment of p62 (ubiquitin adaptor protein) and LC3 (autophagosome marker), resulting in autophagic activation (41, 42) (Figure 3). During multiplication within the cytosol, *Shigella* outer membrane protein VirG (IcsA) accumulates at one pole of the bacterial surface. There, VirG recruits and activates N-WASP, which subsequently recruits and activates the Arp2/3 complex, thereby inducing actin polymerization and bacterial motility within the cell (8–10). At this stage, VirG is recognized by the host autophagy protein Atg5, a protein essential for autophagosome maturation, resulting in *Shigella* uptake by autophagosomes. However, *Shigella* prevents autophagic recognition by delivering the IcsB T3SS effector. At an early stage of infection, IcsB binds to and recruits Toca-1, which is required for efficient formation of actin polymerization, around intracellular bacteria, and IcsB–Toca-1 prevents the recruitment of LC3 (43, 44). Furthermore, at the later stage of infection, IcsB plays a pivotal role in camouflage against autophagic recognition (45). IcsB and Atg5 interact with the same region on VirG, but the affinity of IcsB for VirG is stronger than that of Atg5; therefore, IcsB competitively inhibits the VirG–Atg5 interaction, masking the target VirG protein from autophagic recognition (45) (Figure 3). Consistent with this, the *Shigella* Δ*icsB* mutant is trapped in the autophagosome and delivered for lysosomal degradation (45). Intriguingly, IcsB has cholesterol binding region, and its ability is involved in evading autophagic recognition without affecting IcsB–VirG binding (46). In *Shigella* infection, ubiquitin-dependent selective autophagy is also triggered, but the bacteria evade ubiquitin recognition. Although *Shigella* WT is not targeted by ubiquitin, LRSAM1 (a mammalian LRR-containing RING E3 ligase), which itself acts as a bacterial recognition molecule, localizes to the *Shigella* Δ*icsB* mutant, and LRSAM1-mediated bacterial ubiquitylation has been observed *in vitro* (44). LRSAM1 recognition can trigger ubiquitin-dependent selective autophagy, resulting in restriction of bacterial replication (47). In addition, another host factor septin, which is GTP-binding protein, assembles at sites of VirG-induced actin polymerization and forms cages that surrounding bacteria, and prevents inter- and intracellular movement, thereby targeting by autophagy and restricting bacterial proliferation (48). A recent report showed that the ubiquitin adaptors p62 and NDP52 target *Shigella* for autophagy in an actin polymerization- and septin-dependent manner, and that the *Shigella* Δ*virG* mutant reduces the recruitment of p62 and NDP52 (49). The *Shigella* Δ*icsB* mutant increases septin-cage formation and the recruitment of ubiquitin, p62, and NDP52. Therefore, although VirG-mediated actin polymerization is targeted by septin- and ubiquitin-dependent selective autophagy, the IcsB–VirG interaction prevents the recruitment of ubiquitin, p62, and NDP52 around bacteria (49) (Figure 3).

## Membrane Trafficking

Intracellular trafficking of membranes and proteins is essential for maintenance of epithelial homeostasis and barrier function, and also acts as a host defense system against bacterial pathogens. In eukaryotic cells, intracellular trafficking system can be divided into two pathways, endocytosis and the secretory pathway. During endocytosis, which internalizes extracellular molecules or delivers plasma membrane proteins to specialized sites, the cargo is sorted in the early endosome and further transported to Golgi, endoplasmic reticulum (ER), or late endosomes and lysosome for degradation, whereas some cargo proteins are transported back to the plasma membrane via recycling endosomes. On the other hand, the secretory pathway, which produces molecules such as antimicrobial peptides, growth factors, cell surface receptors, and cytokine secretion, transport molecules from the ER to the plasma membrane through the Golgi.

**Figure 4:**
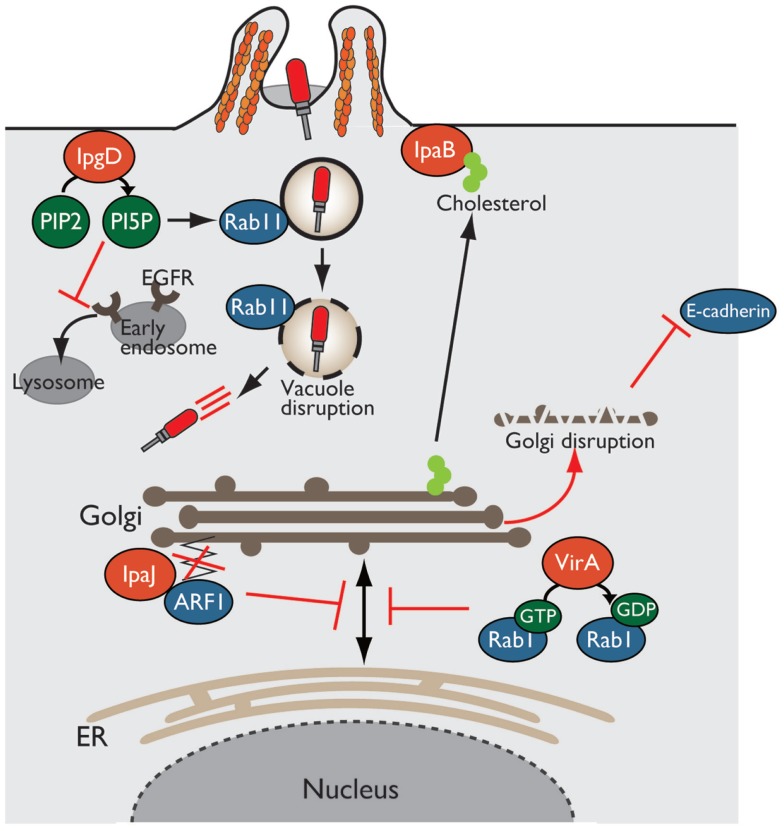
***Shigella* alters host intracellular trafficking**. *Shigella* exploits and alters intracellular trafficking to manipulate the host defense system. PI5P produced by IpgD recruits Rab11 to the bacterial entry site and promotes vacuolar membrane disruption. Production of PI5P by IpgD further relocates EGFR in early endosomes, resulting in blocking lysosomal degradation of EGFR and sustaining PI3K–Akt survival pathway. VirA and IpaJ target and inactivate Rab1 and ARF1, respectively, thereby preventing intracellular trafficking and Golgi disruption. IpaB binds to and relocates cholesterol from Golgi to bacterial entry sites, resulting in Golgi fragmentation.

Exocytosis, endocytosis, phagocytosis, and cytokine secretion play essential roles as host defense systems for eliminating invading bacterial pathogens. However, many intracellular bacterial pathogens, such as *Shigella*, *Salmonella*, and *Legionella*, hijack and exploit host intracellular trafficking system to enter host cells, evade subsequent phagocytic destruction, and establish their safety replicative niche for their survival and proliferation (7, 50, 51). *Shigella* enter non-phagocytic cells by delivering a coordinated set of T3SS effector proteins that trigger actin cytoskeleton or plasma membrane remodeling, and promote subsequent bacterial uptake by host cells. Soon after internalization, the bacteria are engulfed within a membrane-bound vacuole that is derived from the host plasma membrane. After that, *Shigella* disrupt and escape from this vacuole and replicate within the cytoplasm (7). Vacuolar disruption or modulation by intracellular bacteria is related to the host membrane trafficking system, which is tightly regulated by small GTPases of the Rab and ARF families. The small GTPases act as molecular switches that cycle between the GTP-bound active form and GDP-bound inactive form, catalyzed by two classes of regulatory proteins, guanine nucleotide exchange factors (GEFs) and GTPase-activating proteins (GAPs). GEFs exchange GDP for GTP to activate GTPases, whereas GAPs inactivate GTPases by promoting hydrolysis of GTP to GDP (52). Rab and ARF GTPases play an important role in resisting bacterial infection, such as secretion of cytokines and antimicrobial peptides, maintenance of epithelial barrier integrity, and maturation of phagosomes into lysosomes to degrade entrapped bacteria. Therefore, *Shigella* target Rab GTPases and interfere with host membrane trafficking by delivering T3SS effector proteins (Figure 4).

In *Shigella* infection, the endocytic pathway is essential event for bacterial invasion, vacuolar rupture, and spread to neighboring cells. Using a high-content siRNA screening, Mellouk et al. recently demonstrated that *Shigella* targets and recruits Rab11, a component of host recycling endosomes, to disrupt the vacuole and efficiently escape from it by delivering the IpgD effector (53) (Figure 4). As described before, IpgD is a phosphoinositide phosphatase that produces PI5P, which is important for vesicular trafficking. In cells infected with *Shigella* Δ*ipgD* or phosphatase-inactive Δ*ipgD*/*ipgD* C438S complemented mutant, Rab11 is not recruited to the site of bacterial invasion, and vacuolar rupture is delayed; however, this deficiency is rescued by the complementation of Δ*ipgD/ipgD* WT or WT *Shigella* infection, highlighting the requirement for the phosphoinositide phosphatase activity of IpgD. Furthermore, vacuole disruption by *Shigella* is delayed in Rab11 knockdown epithelial cells without affecting bacterial invasion (53). Therefore, *Shigella* targets Rab11 to disrupt the vacuole and allow bacterial escape into the cytoplasm and promote further bacterial replication and dissemination.

In addition, as mentioned above briefly, IpgD subverts host intracellular trafficking to promote host-cell survival for bacterial prolonged colonization (34). The production of PI5P by IpgD at bacterial entry sites recruits active EGFR, which is required for the PI3K–Akt survival signaling pathway. An elevated level of PI5P at the plasma membrane, due to the activity of IpgD, alters EGFR trafficking and relocates it to early endosomes from late endosomes or lysosomes, thereby blocking lysosomal degradation of EGFR and sustaining EGFR–PI3K–Akt survival signaling activation (34) (Figure 4).

The Golgi apparatus is a central organelle involved in protein or lipid transport in eukaryotic cells. The Golgi processes and sorts proteins made by the ER, and transport them to various destinations, such as the plasma membrane and lysosome. Therefore, defects in Golgi function result in the loss of intracellular trafficking, including secretion of antimicrobial peptides and cytokines, and transport of epithelial junction proteins that are essential for epithelial barrier function against bacterial infection. Intriguingly, *Shigella* disrupts the Golgi apparatus by inhibiting vesicle trafficking in infected epithelial cells in a T3SS-dependent manner. Of note, three *Shigella* effectors, VirA, IpaJ, and IpaB, play important roles in disrupting the Golgi apparatus by targeting host intracellular trafficking (Figure 4).

Recent reports have shown that VirA and IpaJ, which have specific enzymatic activities targeting Rab and ARF GTPases, respectively, dampen host antibacterial defense systems by inhibiting the host secretory pathway. VirA (a homolog of EPEC EspG), which has TBC (homologous catalytic domain of GAP for Rab GTPase)-like GAP activity, preferentially targets and catalyzes GTP hydrolysis in Rab1, which localizes in the ER and mediates ER-to-Golgi trafficking (54). VirA inactivates Rab1, inhibits ER-to-Golgi transport of Rab1-containing vesicles, and disrupts the Golgi apparatus in host epithelial cells (Figure 4). Because Rab1 is important for autophagosome formation, Rab1 inactivation by VirA counteracts antibacterial autophagy. Of note, infection by a *Shigella* Δ*virA* mutant or GAP-inactive Δ*virA/virA-RQ* complemented mutant increases autophagosome formation and reduces the number of colonizing bacteria relative to that of WT *Shigella* in human epithelial cell lines. Thus, TBC-like GAP activity of VirA contributes to bacterial escape from autophagy and intracellular survival by blocking intracellular trafficking through Rab1 GTPase inactivation (54).

A bioinformatics approach revealed that IpaJ belongs to the cysteine protease family, whose members have Cys–His–Asp catalytic triad residues (55, 56). Further yeast genetic screening and mass spectrometry analysis demonstrated that IpaJ specifically cleaves the *N*-myristoylated glycine from ARF1 (Figure 4). *N*-myristoylation is an essential fatty-acid modification that is involved in protein localization, signal transduction, autophagosome maturation, and organelle function. Because *N*-myristoylation of ARF1 is essential for binding to the Golgi membrane, IpaJ-mediated cleavage induces the release of ARF1 from Golgi and its disruption, thereby inhibiting intracellular trafficking, including cytokine secretion and signal transduction (55, 56).

Another effector, IpaB, also disrupts the Golgi apparatus by targeting lipid trafficking, and thereby reduces epithelial barrier function. IpaB binds to and redirects cholesterol at the bacterial entry site from the Golgi apparatus, which depletes the lipid of Golgi and induces its fragmentation (Figure 4). The Golgi apparatus fragmentation further induces tubulation of the Rab11-positive compartment and reorganizes the recycling endosome, which is involved in trafficking of E-cadherin to adherence junction, resulting in epithelial junction disruption (57).

As described above, membrane and protein trafficking, such as the phagosome component, epithelial barrier components, antimicrobial peptides, cytokines, and cell surface receptors, is a major host defense system against bacterial infection. Therefore, *Shigella* blocks and alters host intracellular trafficking by delivering subsets of effectors that promote infection.

## Manipulation of Host Innate Immunity

**Figure 5:**
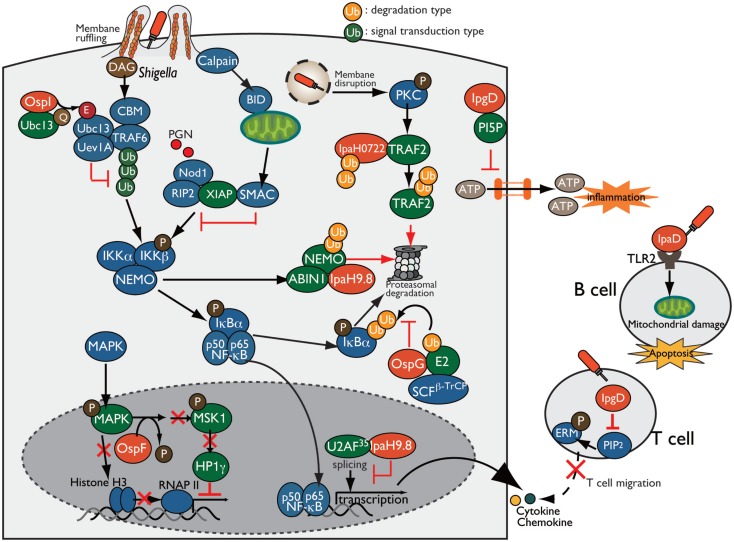
***Shigella* manipulates the host innate and adaptive immune response**. *Shigella* manipulates host inflammatory responses by delivering a subset of T3SS effectors, including IpgD, OspI, OspG, OspF, and IpaH. PI5P, generated by IpgD, prevents ATP release-dependent inflammation through hemichannels. OspG binds to ubiquitin and ubiquitinated E2 proteins and prevents IκBα ubiquitylation, which is required for NF-κB activation. OspI deamidates and inactivates Ubc13, resulting in inhibition of TRAF6 ubiquitination. IpaH0722 and IpaH9.8 target TRAF2 and NEMO, respectively, for ubiquitination, and undergo proteasome degradation. In addition, IpaH9.8 further targets U2AF35, preventing its participation in the splicing reaction via an unidentified bacterial factor, *Shigella* induces calpain-dependent BID activation, which, in turn, releases mitochondrial SMAC to antagonize XIAP-mediated inflammation. IpgD-mediated hydrolysis of PIP2 inactivates ERMs of T cells, which crosslink actin filaments with the plasma membrane, thereby preventing T cell migration. *Shigella* induces B cell death in both invaded and non-invaded cells. In non-invaded cells, the T3SS needle-tip protein IpaD interacts with TLR2 on B cells and triggers mitochondrial damage, which eventually induces apoptosis.

Once *Shigella* invades and replicates within host cells, the innate immune system quickly senses PAMPs or DAMPs, and transmits various alarm signals to the rest of the immune system, and ultimately triggers inflammation. Inflammation, which is accompanied by inflammatory cytokine secretion, neutrophil recruitment, and massive tissue destruction, is the hallmark of the host innate immune response that eventually restricts and eliminates bacterial infection. However, many bacterial pathogens, including *Shigella*, deliver a subset of T3SS effectors that manipulate host innate immune responses, thereby promoting bacterial colonization and survival (2) (Figure 5).

### ATP Release

During bacterial infection, various molecules are released from damaged or stressed cells as DAMPs (e.g., ATP, uric acid, and membrane remnants), which trigger inflammatory immune responses. For example, infection of intestinal epithelial cells with enteric bacterial pathogens, such as *Shigella, Salmonella*, and EPEC, induce connexin hemichannel-dependent ATP release, which acts as an endogenous danger alarm against bacterial infection and triggers inflammatory responses (58). Hemichannel-dependent ATP release is a common host defense system against enteric bacterial infection. To counteract ATP-dependent inflammation, *Shigella* blocks ATP release by delivering IpgD to epithelial cells. IpgD blocks hemichannels and prevents ATP release through production of PI5P, which regulates hemichannel opening, thereby dampening ATP-dependent inflammation (Figure 5). Consistent with this, in both *in vitro* and *in vivo* infections, the *Shigella* Δ*ipgD* mutant causes elevated ATP release, severe intestinal inflammation, and mucosal damage, illustrating the key role of IpgD in preventing ATP-dependent inflammation (58).

### Manipulation of NF-κB Signaling

During bacterial infection, the transcription factor NF-κB, the master regulator of pro-inflammatory cytokines, plays a major role in mediating an inflammatory signaling pathway that triggers a wide range of host inflammatory responses. In response to bacterial infection, several intracellular and extracellular stimuli activate signal transduction cascades, and NF-κB is translocated into the nucleus, where it promotes the transcription of target genes (59). In particular, ubiquitination of signaling factors is prerequisite for regulation of NF-κB activity; therefore, many bacterial pathogens, including *Shigella*, inhibit NF-κB activation by targeting signaling factors and altering signal transduction, thereby dampening inflammation, in order to promote infection (60).

Invasion of epithelial cells by *Shigella* produces membrane ruffles by remodeling the actin cytoskeleton around the bacterial entry site. Aberrant membrane ruffles, which protrude from the bacterial entry site and are accompanied by diacylglycerol (DAG) production, are sensed as DAMPs by the host innate immune system and trigger the activation of the DAG-CBM (CARMA-BCL10-MALT1)-TRAF6-NF-κB pathway. However, *Shigella* delivers OspI via the T3SS into the host cells; this factor targets and deamidates UBC13 (converts Gln-100 to Glu-100), an E2 required for TRAF6 E3 activity, resulting in abolition of its E2 activity and thereby interfering with the DAG–CBM–TRAF6–NF-κB pathway (61) (Figure 5).

Following membrane ruffling, *Shigella*-mediated vacuolar membrane ruptures are also recognized as DAMPs, triggering an additional alarm-signaling pathway via recruitment and activation of PKC, ultimately leading to the activation of the PKC–NF-κB pathway. To counteract PKC–NF-κB activation, *Shigella* delivers IpaH0722, one of the IpaH family of E3 ubiquitin ligase effectors. IpaH0722 dampens the acute inflammatory response by ubiquitination of TRAF2, a molecule downstream of PKC, thereby preferentially inhibiting PKC-mediated NF-κB activation (62) (Figure 5).

During *Shigella* invasion of epithelial cells, Nod1 recognizes PGNs released from *Shigella* as PAMPs, triggering the Nod1–RIP2 pathway and activating the downstream mitogen-activated protein kinase (MAPK) and NF-κB signaling pathways. *Shigella* delivers IpaH9.8, an IpaH family E3 ubiquitin ligase effector, which preferentially prevents the Nod1-dependent NF-κB activation. IpaH9.8 interacts with NEMO/IKKγ, an essential component of the IKK kinase complex, and targets NEMO for ubiquitination. IpaH9.8 also interacts with ABIN-1, an ubiquitin-binding adaptor protein, to further promote polyubiquitylation of NEMO. Ubiquitinated NEMO by IpaH9.8 subsequently leads to proteasomal degradation, thereby diminishing NF-κB activation (63) (Figure 5). The activity of this E3 ligase effector during *Shigella* infection contributes to bacterial colonization in a mouse lung infection models (63).

Upon Nod1 activation, the X-linked inhibitor of apoptosis protein (XIAP) plays a crucial role in activating Nod1-dependent inflammatory responses. XIAP interacts with RIP2 and facilitates NF-κB activation, ultimately leading to pro-inflammatory gene transcription. A recent report showed that *Shigella* evades XIAP-mediated inflammation by actively releasing mitochondrial SMAC (36). *Shigella* infection triggers calpain activation and processes and activates BH3-only protein BID, which then translocates to the mitochondria and induces the release of SMAC. Released SMAC binds to XIAP, and inhibits XIAP-mediated NF-κB activation without inducing mitochondrial damage or cell death (Figure 5). Although it remains unknown whether BID-mediated release of SMAC by *Shigella* depends on a T3SS effector, *Shigella* uniquely exploits mitochondrial function to block the host innate immune response while avoiding cell death (36).

In addition, *Shigella* delivers another effector, OspG, which shares sequence similarity with mammalian serine/threonine kinases and inhibits NF-κB activation (64). OspG binds to ubiquitin and ubiquitinated E2s, which are required for phospho-IκBα ubiquitination by an E3 ligase such as SCF^β-TrCP^, and formation of this OspG–E2-ubiquitin complex promotes OspG kinase activity and increases the prevention of NF-κB activation (64–67) (Figure 5).

### Epigenetic Regulation of Immune Gene Expression

Because the host transcriptional program plays a pivotal role in triggering inflammation and eliminating bacterial infection, *Shigella* overcomes this defense mechanism by delivering a subset of T3SS effector proteins that regulate gene expression and counteract host immune responses. In addition to interfering with NF-κB signaling, as described above, *Shigella* hacks the host immune responses by reprograming the host epigenome to promote infection. The T3SS effector OspF (homolog of *Salmonella* SpvC and *Pseudomonas syringae* HopAl1), which has a unique phosphothreonine lyase activity, translocates into the nucleus of epithelial cells, where it irreversibly dephosphorylates and inactivates MAPKs (Erk and p38) through beta-elimination of the phosphate group (68, 69). Inactivation of MAPK by OspF further blocks downstream phosphorylation of histone H3 at Ser10 at the promoters of a subset of innate immune genes, such as IL-8, and promotes chromatin condensation, resulting in repression of transcription by masking NF-κB binding sites (68) (Figure 5). In addition to histone modification, OspF also alters the activity of the chromatin reader heterochromatin protein 1 (HP1) and represses host gene expression during *Shigella* infection (70). Although phosphorylated HP1γ at Ser83 proteins positively regulate euchromatin (a transcriptionally active state) and activate transcription, OspF inactivates Erk and consequently reduces the activity of the downstream kinase MSK1, a kinase for HP1γ at Ser83. As a result of HP1γ dephosphorylation, HP1γ dissociates from sites of transcriptional activation at OspF-target genes such as IL-8 (Figure 5). Consistent with this, the *Shigella* Δ*ospF* mutant increased the level of HP1γS83 phosphorylation relative to WT *Shigella*-infected cells (70). Therefore, OspF manipulates host transcriptional response via two epigenetic modifications: (i) decreasing the level of phosphorylated histone H3, and (ii) altering the activity of HP1 through dephosphorylation of MAPK, which contributes to downregulation of host inflammatory responses.

In addition to OspF, *Shigella* delivers IpaH9.8 to regulate pro-inflammatory gene expression. IpaH9.8 is translocated into the nucleus of epithelial cells, where it binds to U2AF35, an mRNA splicing factor, and inhibits the U2AF35-dependent splicing reaction, enabling the bacterium to dampen expression of numerous genes, including some that encode pro-inflammatory cytokines and chemokines (71) (Figure 5).

## Manipulation of Host Adaptive Immunity

Manipulation of the host innate immune response by *Shigella* is a pivotal survival strategy for promoting infection; however, the interactions between *Shigella* and adaptive immune response, such as T and B lymphocytes, have not been thoroughly investigated. One prominent issue is the lack of appropriate animal infection models that mimic human intestinal infection. Until now, however, several studies have proven the importance of adaptive immunity against *Shigella* infection using a mouse pulmonary infection model that mimics the acute inflammation that occurs during shigellosis. Mice that are genetically deficient in B, T, and NK cells are much more susceptible to *Shigella* infection than WT mice, and T and NK cells play critical roles in clearing *Shigella* (72). Additional studies have shown that CD4^+^ T helper 17 (Th 17) cells, which are predominantly primed in response to *Shigella*, produce IL-17A and eventually restrict secondary *Shigella* infection, indicating the involvement of Th17 cells in adaptive immunity against *Shigella* infection (73). By contrast, antigen-specific CD8^+^ T cells, which are usually required for adaptive immunity against cytosolic bacterial infection, are not primed and involved in adaptive immunity against *Shigella* infection (74). As the precise role and the importance of adaptive immunity against *Shigella* infection have been revealed, several recent studies have shown that host adaptive immunity is targeted and subverted by *Shigella* T3SS effectors (Figure 5).

T cell migration and activation are key events in the induction of antibody- and cell-mediated immune responses against bacterial infection. Recently, the Phalipon group investigated the interaction between T cells and *Shigella* using *in vitro* and *in vivo* approaches, and demonstrated that *Shigella* interferes with adaptive immune responses by targeting T cells. They also found that *Shigella* invades activated CD4^+^ T cells and inhibit chemoattractant-mediated T cell migration by delivering IpgD (75). T-lymphocyte migration toward a chemoattractant depends on the membrane cytoskeleton crosslinkers proteins ERM (ezrin, radixin, and moesin), which are tightly converted between the active and inactive conformation by the concentration of PIP2 at the plasma membrane. Because IpgD is phosphoinositide phosphatase, it subsequently hydrolyzes and decreases the concentration of PIP2 at the plasma membrane, thereby inactivating ERM (Figure 5). In addition, *Shigella* impairs CD4^+^ T cell dynamics within lymph nodes, where adaptive immunity is initiated, in the mouse infection model (76). Therefore, *Shigella* targets T-lymphocytes and inhibits their migration by delivering the T3S effector IpgD, thereby interfering with adaptive immunity.

B lymphocytes are key players in antibody-mediated immunity, and they produce and secrete cytokines that contribute to the antibody-independent immune response against bacterial infection. However, massive T- and B-cell deaths are observed in rectal biopsies of *Shigella*-infected humans, indicating that *Shigella* impairs B cell-mediated immunity (77). To support this notion, a recent report showed that *Shigella* targets B cells and induces cell death in both *Shigella*-invaded and non-invaded cells (78). *Shigella* invades B cells and replicates intracellularly, resulting in B cell death. Furthermore, *Shigella* induces B cell apoptosis via the T3SS needle-tip protein IpaD. IpaD binds to TLR2 on B cells and triggers the loss of mitochondrial dysfunction and apoptotic cell death signaling in non-invaded B cells, eventually helping the bacterium to avoid antibody-mediated immune responses (78) (Figure 5). Therefore, *Shigella* targets T and B cells and manipulates adaptive immunity against *Shigella* infection, thereby preventing antibody-mediated lasting immunity and promoting bacterial infection.

## Conclusion

Although bacterial infection elicits host defense systems that would restrict and eliminate bacteria, many bacterial pathogens have evolved excellent strategies to manipulate host immune responses and enable bacterial colonization. Here, we review the current understanding of how *Shigella* manipulates host immune responses, focusing specifically on T3SS effector-mediated interference with host signaling transduction cascades, alteration of membrane trafficking, and modulation of host-cell death. Our understanding of the molecular basis of the interaction between bacterial effectors and host immune systems has advanced greatly during the past decade. Although accumulating evidence has revealed the importance of manipulation of the host immune system during bacterial infection, our knowledge of bacterial strategy is still in its infancy. Although approximately 50 T3SS effectors of *Shigella* are currently recognized, we have only elucidated the molecular function of one-third of them. As has been shown for other bacterial pathogens, the effector activity and strategies described above are not unique to *Shigella*, but are instead shared as common strategies by many bacterial pathogens. Therefore, the discovery of new *Shigella* strategies that manipulate the host immune system will not only provide a new understanding of how bacteria subvert the host immune system but also facilitate development of new types of antibacterial drugs that target bacterial effectors and bacterial live vaccines to overcome bacterial infections.

## Conflict of Interest Statement

The authors declare that the research was conducted in the absence of any commercial or financial relationships that could be construed as a potential conflict of interest.
